# The Epigenetic Role of Vitamin C in Neurodevelopment

**DOI:** 10.3390/ijms23031208

**Published:** 2022-01-21

**Authors:** Sharna J. Coker, Carlos C. Smith-Díaz, Rebecca M. Dyson, Margreet C. M. Vissers, Mary J. Berry

**Affiliations:** 1 Perinatal & Developmental Physiology Group, Department of Paediatrics & Child Health, University of Otago, Wellington 6242, New Zealand; sharna.coker@postgrad.otago.ac.nz (S.J.C.); becs.dyson@otago.ac.nz (R.M.D.); 2 Centre for Free Radical Research, Department of Pathology and Biomedical Science, University of Otago, Christchurch 8140, New Zealand; carlos.smith-diaz@postgrad.otago.ac.nz

**Keywords:** ascorbate, epigenetic programming, maternal nutrition, neurodevelopment, ten-eleven translocation methylcytosine dioxygenases, TET enzymes, vitamin C

## Abstract

The maternal diet during pregnancy is a key determinant of offspring health. Early studies have linked poor maternal nutrition during gestation with a propensity for the development of chronic conditions in offspring. These conditions include cardiovascular disease, type 2 diabetes and even compromised mental health. While multiple factors may contribute to these outcomes, disturbed epigenetic programming during early development is one potential biological mechanism. The epigenome is programmed primarily in utero, and during this time, the developing fetus is highly susceptible to environmental factors such as nutritional insults. During neurodevelopment, epigenetic programming coordinates the formation of primitive central nervous system structures, neurogenesis, and neuroplasticity. Dysregulated epigenetic programming has been implicated in the aetiology of several neurodevelopmental disorders such as Tatton-Brown-Rahman syndrome. Accordingly, there is great interest in determining how maternal nutrient availability in pregnancy might affect the epigenetic status of offspring, and how such influences may present phenotypically. In recent years, a number of epigenetic enzymes that are active during embryonic development have been found to require vitamin C as a cofactor. These enzymes include the ten-eleven translocation methylcytosine dioxygenases (TETs) and the Jumonji C domain-containing histone lysine demethylases that catalyse the oxidative removal of methyl groups on cytosines and histone lysine residues, respectively. These enzymes are integral to epigenetic regulation and have fundamental roles in cellular differentiation, the maintenance of pluripotency and development. The dependence of these enzymes on vitamin C for optimal catalytic activity illustrates a potentially critical contribution of the nutrient during mammalian development. These insights also highlight a potential risk associated with vitamin C insufficiency during pregnancy. The link between vitamin C insufficiency and development is particularly apparent in the context of neurodevelopment and high vitamin C concentrations in the brain are indicative of important functional requirements in this organ. Accordingly, this review considers the evidence for the potential impact of maternal vitamin C status on neurodevelopmental epigenetics.

## 1. Introduction

Most animals synthesise vitamin C (ascorbate, ascorbic acid) in the liver, and provide a steady supply to the body through the circulation. However, humans and a few other species including other primates and guinea pigs have lost this capability due to loss-of-function mutations in the gene encoding gulonolactone oxidase, the terminal enzyme in the vitamin C biosynthetic pathway [1,2]. Consequently, humans are entirely dependent on dietary sources of vitamin C to maintain tissue concentrations. Inadequate dietary intake results in scurvy, a potentially fatal disease that manifests at plasma vitamin C concentrations <11 umol/L in humans, guinea pigs and other animals unable to endogenously synthesise vitamin C [3,4]. Although cases of clinical scurvy are now rare in the developed world, suboptimal vitamin C status or hypovitaminosis C, defined as a plasma concentration <23 umol/L, is surprisingly common, affecting approximately 15% of the adult population [3,4,5]. This prevalence is markedly higher in specific subgroups such as pregnant women, smokers and low-socioeconomic classes [3,4,6,7].

The importance of vitamin C during pregnancy to support normal fetal growth and development has been recognised for decades. As early as 1938, it was reported that the fetus “acts as a parasite on the mother’s vitamin C pool” following the observation that concentrations of vitamin C in the umbilical cord plasma of newborn infants were 2–4 fold higher than that of their mothers at the time of delivery [8]. A similar phenomenon is observed in guinea pigs, with plasma vitamin C concentrations in newborn pups being twice as high as those in dams [9]. Guinea pig fetal vitamin C concentrations are significantly elevated at gestational day (GD) 45 (term delivery ~69 days) [10], which coincides with the peak of overall brain growth [11]. Suboptimal vitamin C status in prenatal (beginning in the first trimester until birth) [12], or early postnatal life (6–7 days old) [13] drastically impairs hippocampal development and induces oxidative stress in the developing guinea pig brain [14,15]. Additionally, mice lacking brain vitamin C transporters die soon after birth with severe cerebral haemorrhages [16]. These findings suggest that vitamin C plays a central role in the developing brain.

Vitamin C has many established functions in the nervous system. It acts as a co-factor for a plethora of different enzymes which are involved in diverse processes including epigenetic regulation, the biosynthesis of catecholamine neurotransmitters and hormones, collagen production and angiogenesis. Another function of the micronutrient is scavenging reactive oxygen species (ROS). Given the diverse roles of vitamin C in human biochemistry, the scope of this review is the role of vitamin C as an epigenetic regulator during neurodevelopment. This is a relatively narrow field, but one with broad implications for our understanding of health and disease.

## 2. Regulation of Vitamin C In Vivo

Vitamin C is a diacid-ascorbic acid. At a neutral pH, the nutrient is present almost exclusively (>99.9%) in the ascorbate monoanion form. Ascorbate is an electron donor that is readily oxidised firstly to the ascorbyl radical and then to dehydroascorbic acid [1]. Dehydroascorbic acid is an unstable molecule that rapidly hydrolyses at neutral pH, unless it is reduced back to ascorbate by glutathione-dependent cellular reducing systems [17,18]. As an electron donor, ascorbate is indispensable for a wide range of biochemical processes. 

Tissue vitamin C homeostasis is regulated by the sodium-dependent vitamin C transporters (SVCT) 1 and 2 that actively co-transport ascorbate with sodium across the cell membrane [19,20]. Dietary absorption is mediated by SVCT1 located in the small intestine [19,21] and the distribution from the bloodstream to target organs is mediated by SVCT2, a transporter that is widely expressed in all tissues including the choroid plexus [19,22]. Dehydroascorbic acid can compete with glucose and be taken into cells via the glucose transporters (GLUTs). However, under normal physiological conditions, there is very little dehydroascorbic acid present, with concentrations 1000-fold less than normal glucose, resulting in relatively little uptake via the GLUT transporters [23]. 

**Figure 1:**
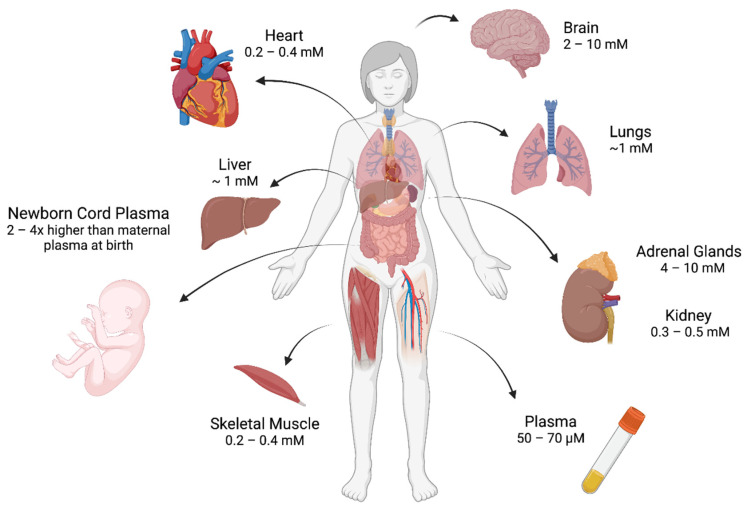
Tissue Distribution of Vitamin C. Vitamin C is present at different concentrations in different tissues. Notably, the concentration of vitamin C is particularly high in the brain and adrenal glands [19], with the newborn retaining higher plasma concentrations of vitamin C that its mother at the time of birth [8]. These observations highlight the importance of understanding the role of vitamin C during neurodevelopment. Figure created with Biorender.com using published data [19].

Vitamin C displays a diverse distribution pattern across the body, with homeostatic concentrations ranging from 200 μM in the heart to ~10 mM in the brain and adrenal glands in humans (Figure 1) [19,24,25]. Remarkably, the brain can maintain this high vitamin C content even during periods of chronic dietary deficiency, emphasising the vital role of vitamin C in the brain [19,26,27,28]. The main route of entry into the brain is through SVCT2 transporters expressed on the luminal side of the choroid plexus epithelium [22,29,30]. The highest concentrations of brain vitamin C are found in the cerebral cortex, the hippocampus and the cerebellum where SVCT2 expression is enriched [25,26,30,31].

The developing fetus accesses vitamin C via SVCT2 transporters in the placenta and therefore relies on a sufficient maternal supply for the entire gestational period [16,21,32]. The discovery that newborn human infants display higher plasma vitamin C concentrations (as measured in cord plasma at birth) than their mothers [6,8,33], suggests that the nutrient is imperative for normal fetal development. However, this preferential transport of vitamin C into the fetus does not fully compensate for poor maternal vitamin C status. In humans, low maternal vitamin C status during pregnancy also corresponds with low concentrations of vitamin C in the cord plasma of newborn infants [6]. Additionally, low vitamin C status in mothers has been associated with pregnancy complications [34] such as an increased risk of preeclampsia [34,35], preterm delivery [36], and low birth weight infants [37,38]. Vitamin C supplementation during pregnancy to improve these outcomes has produced varied results [39,40,41,42]. However, specific subgroups such as women with low dietary vitamin C intakes, smokers, and diabetics, are often excluded or not discussed, highlighting the demand for further research.

Guinea pig studies indicate that plasma vitamin C concentrations in newborn pups are also significantly higher than in the mother, when the mother has an optimal dietary intake of vitamin C [9]. Guinea pig data also demonstrates that the mother’s vitamin C requirement is prioritised, and preferential placental transport is overridden during periods of chronic vitamin C deficiency [9]. As has been observed in human pregnancy, low vitamin C status in guinea pigs has been associated with poor reproductive performance and worse pregnancy outcomes as measured by an increased number of aborted and reabsorbed fetuses and poor fetal growth in mid gestation [43]. Another guinea pig study reported similar findings with low maternal vitamin C concentrations leading to reduced fetal body, brain, and placental weights at GD 45 (second trimester). However, these effects were transient as weights had normalised by GD 56 (third trimester) [10]. At term (~GD 69), the birth weights of pups born to dams with low vs. sufficient vitamin C status were also comparable [9]. These findings suggest that vitamin C is particularly important during the earlier stages of fetal development.

## 3. Fetal Programming

In the late 1980’s Barker et al. demonstrated a significant association between decreased birth size and cardiovascular disease in adulthood that gave rise to the Developmental Origins of Health and Disease (DOHaD) phenomenon [44,45]. Barker’s hypothesis states that insufficient nutrition in utero leads to disproportionate fetal growth and “programs” metabolic characteristics that predispose the fetus to chronic disease in later life [46,47]. A mechanistic construct underpinning the DOHaD phenomenon is the Predictive Adaptive Response (PAR) model that postulates that the developing fetus forms an impression of its future postnatal world via environmental cues including maternal nutrient availability, and adapts accordingly [48,49]. When the predicted and actual postnatal environments differ, previously beneficial adaptations can become maladaptive [50,51,52]. When considering maternal undernutrition for instance, the fetus may undergo metabolic adaptations in utero that encourage the thrifty utilisation of limited energy sources such as increased insulin sensitivity [50,51,52]. Predictive adaptive responses, through which one genotype can give rise to a range of phenotypes in response to different environments are thought to occur via epigenetic mechanisms that modulate gene expression such as DNA methylation and histone modification [53].

## 4. The Fundamentals of Epigenetics

A modern definition of epigenetics is “the study of changes in gene function that are mitotically and/or meiotically inherited without changes in DNA sequence, but with chemical modifications of DNA, or of the structural and regulatory proteins that are bound to it” [54,55]. While there are many complex mechanisms that regulate gene expression, one of the most important and widely studied processes is chromatin remodelling via DNA or histone methylation. Changes in chromatin states may influence gene expression, imparting cell-specific transcription programs that allow cells to become phenotypically different from each other [56]. 

During DNA methylation, a methyl group is added at C5 of cytosine by the DNA methyltransferase (DNMT) enzymes, generating 5-methylcystosine (5 mC). CpG islands are by far the most heavily methylated regions of the mammalian genome [57]. Most CpG islands lie within gene promoter regions where they function as transcription start sites [57,58]. The established view of DNA methylation had been that it was associated with transcriptional repression. With the discovery in 2009 of the TET enzymes, a family of DNA demethylase enzymes, DNA methylation is now viewed as a dynamic and nuanced process [56,59,60]. Chemical modification of amino acids in histone tails by methylation, acetylation, phosphorylation, or ubiquitination also results in chromatin remodelling. These modifications affect the accessibility of DNA for transcription [61]. Lysine methylation is a major histone modification and can act as a marker for both open or closed chromatin states [62,63,64]. Lysine residues are methylated by the histone lysine methyltransferases, and demethylated by the histone lysine demethylases [65]. Non-coding RNAs can also regulate transcription by interacting directly with chromatin modifiers or by intercepting transcription products prior to translation. MicroRNAs for example bind to recently transcribed mRNA molecules and recruit a silencing complex that degrades the mRNA before translation can take place [66]. This represents a third general process underpinning the epigenetic regulation of gene expression.

## 5. Epigenetic Programming in Embryonic Development

**Figure 2:**
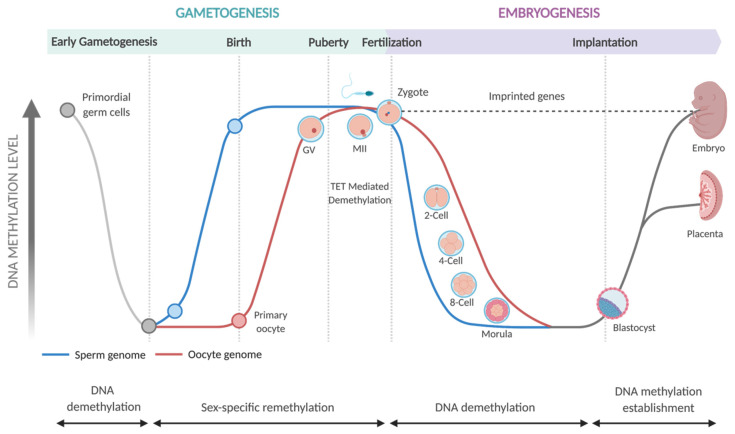
Changes in DNA Methylation during Mammalian Development. See text above for further details. Figure adapted from the templates library available at Biorender.com accessed on 8 December 2021.

The epigenome is established primarily in utero. DNA methylation patterns during gametogenesis and embryogenesis are remarkably dynamic (Figure 2). Methylation changes are initially observed during the development of primordial germ cells in embryos. Highly methylated primordial germ cells undergo a wave of global demethylation during gametogenesis [67]. Global methylation is then restored in developing germ cells. However, this occurs at different times in male and female embryos. In male embryos, developing gametes undergo extensive re-methylation before birth. In contrast, the methylation of female gametes is progressively restored in the period between birth and the onset of puberty [67].

Following fertilization, global DNA demethylation occurs in both the maternal and paternal pronuclei, giving rise to the totipotent zygote [68,69]. DNA demethylation of the paternal pronucleus happens rapidly, within four hours of fertilization and before DNA replication has begun. For this reason, demethylation of paternally-derived DNA is considered ‘active’ and is driven by TET-mediated catalysis, whereby methylated cytosines are actively removed and replaced with unmethylated cytosines in a replication-independent process [69,70,71]. In contrast, the demethylation of maternally derived DNA occurs more gradually and is said to be ‘passive’. Passive DNA demethylation occurs when DNA methylation patterns fail to be maintained following DNA replication. Over several cell divisions, this results in a gradual depletion of 5mC [69,71]. 

During morphogenesis, the totipotent zygote transforms into two distinct cell lines, the pluripotent inner cell mass and the trophectoderm, that form the primitive mammalian embryo. The inner cell mass eventually forms the embryo proper and the trophectoderm forms the placenta. Following implantation, the inner cell mass differentiates into the diverse assortment of cells such as myocytes, neurons, and epithelia that will form the tissues of the fetus. This remarkable process occurs in a controlled and stepwise fashion whereby individual cells are ‘programmed’ to begin forming the organs they are destined to become [72]. It is at this point, when the fetus is first beginning to take shape, that de novo methylation patterns become established and the new epigenome is actively maintained [73]. It is also around this time that X chromosome-inactivation is committed in female embryos by extensive CpG methylation [60]. These early cellular differentiation events represent a critical period of plasticity during which the developing organs and biological systems are highly malleable and thus susceptible to environmental perturbations such as nutritional availability [74].

## 6. Nutriepigenomics in Embryonic Development

Nutriepigenomics refers to the study of the effects of nutrients on health and development, as mediated through modulation of the epigenome. A famous example of nutriepigenomics is the immature honeybee that transforms into a queen or a worker bee, two very different phenotypes with identical genotypes, depending on whether the bee is fed royal jelly or pollen [75]. By the same token, human pregnancy signifies a critical window of plasticity during which the normal trajectory of fetal development can be altered by environmental factors like nutritional deficiencies, via epigenetic interactions [74,76].

The best studied nutritional associations with mammalian development include folate, other B vitamins, choline and betaines. It is well-known that adequate intake of these micronutrients during periconception and pregnancy is essential for normal fetal development [73,77]. For instance, folate supplementation for women during early pregnancy reduces the rate of neural tube defects in children [78]. Micronutrients such as folate, choline, and betaine are components of the metabolic pathway that produces S-adenosyl methionine (SAM), the universal methyl donor for the generation of 5mC [77,79]. Unsurprisingly, dietary modulation of these micronutrients during pregnancy can significantly alter methylation patterns in offspring and the phenotypic consequences of disrupted methylation are diverse [80,81,82,83]. For example, the methylation status of the gene encoding the retinoid X receptor-α in newborn infants is associated with the risk of adiposity in later life [80]. Choline deficiency during gestation causes global DNA hypermethylation, as opposed to hypomethylation, through the compensatory upregulation of *Dnmt1* [84], highlighting a complex relationship between nutrition and epigenetic processes. 

Another micronutrient which is critical during pregnancy is vitamin C. As with folate, inadequate vitamin C status during gestation has been linked to a number of developmental conditions including neural tube defects [85,86,87]. Animal studies also highlight the serious issues that can arise for offspring in the context of extreme vitamin C deficiency. Depending on the experimental model, these issues include cardiovascular, pulmonary, hepatic and vertebral defects [88], impaired hippocampal development [12,13] or embryonic death [16,89]. When considering a potential biological mechanism behind these observations, there is now significant interest in the sensitivity of the TET enzymes, which are involved in regulating the genome through DNA demethylation, to vitamin C availability.

## 7. The TET Enzymes

**Figure 3:**
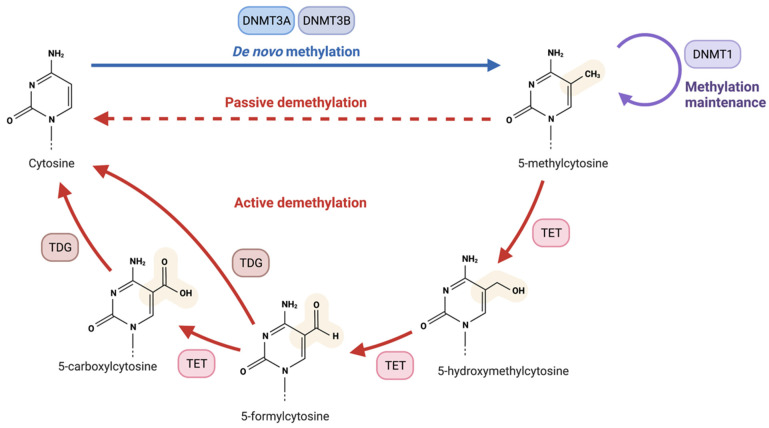
Pathway of DNA Methylation and Demethylation The de novo methylation of cytosine is carried out by DNMT3A and DNMT3B, with methylation being maintained during cell replication by DNMT1. 5-methylcytosine (5mC) is demethylated via two pathways. Passive demethylation occurs due to the failure to maintain methylation after cell division, resulting in the gradual dilution of methylated cytosines. In contrast, the process of active demethylation is catalysed by the TET enzymes (TET1–TET3), which oxidise 5mC to 5-hydoxymethylcytosine (5hmC), 5-formylcytosine (5fC) and 5-carboxylcytosine (5caC). 5fC and 5caC are substrates for thymine–DNA–glycosylase (TDG) which leads to the excision of the oxidised cytosine base. The resulting abasic site is repaired via base excision repair to restore the original cytosine molecule. Figure from Biorender.com.

The TET enzymes (TET 1,2,3) are involved in the oxidation of 5-methylcytosine (5mC) to 5-hydroxymethylcytosine (5hmC), 5-formylcytosine (5fC) and 5-carboxylcytosine (5caC) [90,91,92], crucial steps in the pathway of active DNA demethylation (Figure 3). TET1 was first discovered as an enzyme capable of converting 5mC to 5hmC in 2009 [60] with the discovery of TET2 and TET3 occurring the following year [93]. These enzymes are implicated in diverse epigenetic processes such as cellular differentiation, development and oncogenesis [94,95]. TET mediated demethylation predominantly occurs at regulatory regions of DNA such as promoters and enhancers [96], and TET loss-of-function is associated with hypermethylation, increased genomic instability and the development of haematological cancers [97].

The TET-generated intermediate 5hmC is enriched in tissue specific genes, particularly at enhancers and gene bodies [98,99]. In contrast, 5hmC is underrepresented at intergenic regions of the genome [99]. 5hmC is also highly enriched in genomic areas marked by H3K4me1 (which is associated with active transcription sites) [99]. Collectively, these observations suggest that 5hmC is a marker of active transcription.

The TETs are Fe^2+^ and 2-oxoglutarate dependent dioxygenases (2-OGDDs). These enzymes require O_2_, 2-oxoglutarate (2-OG), Fe^2+^ and, typically, vitamin C for catalytic activity [100,101,102,103,104,105]. The enzymes in this family share a double-stranded β-helix (DSBH) fold containing a catalytic domain containing a non-heme iron bound to a triad of amino acid residues and a separate 2-oxoglutarate binding site [106]. The hydroxylation mechanism employed by all three TET enzymes is characteristic of the 2-OGDDs. This mechanism involves the binding of 2-OG and oxygen to iron followed by the binding of the main substrate [107,108,109]. The TET enzymes generate a highly reactive ferryl-oxo species during the catalytic cycle and utilise this reactive intermediate to abstract a hydrogen from the normally stable C-H bond in 5mC, thereby allowing for hydroxylation to occur [109]. The net effect of this mechanism is the incorporation of one oxygen atom from molecular oxygen into the substrate and the other into succinate–which is released as a product of the reaction.

## 8. Regulation of the TET Enzymes and Vitamin C Dependency

TET activity is highly responsive to substrate and co-factor availability and the enzymes are variously affected by hypoxia, iron availability and the relative concentrations of the Krebs cycle metabolic intermediates fumarate and succinate [110,111]. In recent years, vitamin C has emerged as a potent regulator of TET activity, increasing TET enzyme function in a number of different contexts (Table 1). In vitro studies using the purified catalytic domain of TET2 specifically show that vitamin C promotes an 8-fold increase in the rate of hydroxymethylation [101]. Notably, the authors of this study found that TET2 catalytic activity could not be stimulated by other biological reducing agents such as glutathione, NADPH, L-cysteine, spermidine, vitamin B1 or vitamin E [101]. In contrast to this finding, Hore et al. have shown that some other reducing agents can also promote TET activity in vitro–but still not as effectively as vitamin C [112]. These findings suggest a specific biological role for vitamin C in promoting TET activity [113].

The relationship between vitamin C and TET activity has recently attracted attention in the context of leukaemia research, where vitamin C supplementation is being investigated as a form of epigenetic therapy to enhance TET2 activity in models of leukaemia characterised by decreased TET2 activity [95,121,122,124]. However, despite extensive research, the exact role of vitamin C in the TET catalytic cycle is unknown. Insights from the reaction mechanisms of other 2-OGDD enzymes, notably the functionally related collagen hydroxylases, prolyl-4-hydroxylase and lysyl hydroxylase [113,131], suggest that vitamin C restores the enzyme to its catalytically active Fe^2+^ state when these enzymes undergo an uncoupled reaction in the absence of their substrate [131]. It is possible that the TET enzymes undergo a similar reaction.

There are currently two possible theories which may explain the TET enzymes’ dependency on vitamin C. One idea is that the TET enzymes contain a unique binding site for vitamin C, allowing the molecule to specifically bind and reduce enzyme bound iron at the active site. This mechanism has been proposed to explain the interaction between vitamin C and the related enzyme prolyl-4-hydroxylase [132]. While there is insufficient space within the TET catalytic pocket for ascorbate to bind when all other cofactors are bound [112], it is possible that ascorbate may displace succinate and coordinate to iron at the succinate/2-oxoglutarate binding site. An alternative mechanism proposed by Hore et al. is that vitamin C promotes TET activity, not as a bound co-factor, but rather by converting free Fe^3+^ to Fe^2+^ [112]. Further studies will be required to fully understand the underlying mechanism.

## 9. TET Expression and Function in Mammalian Development

The TETs are expressed with different temporal dynamics during mammalian development, indicating different roles in the regulation of gene expression at different times. TET3 exhibits a high level of expression in oocytes and zygotes [127,129,130] and gene deletion/overexpression studies suggest that it is an important driver of cellular differentiation during mammalian development [128,133]. The differentiation of mouse embryonic stem cells with *Tet3* mutations is skewed towards cells with cardiac mesodermal characteristics. In contrast, ectopic *Tet3* expression favours neural differentiation [128]. TET3 is also thought to be involved in the active demethylation of zygotic paternal DNA following fertilization (as depicted in Figure 2), and knocking down *Tet3* in mice affects the methylation and hydroxymethylation of DNA in the paternal pronuclei [127,129]. 

TET1 and TET2 expression increases during pre-implantation development with high expression in embryonic stem cells [93,118,129] and the inner cell mass [56]. However, TET1 and TET2 again have distinct roles. Knockout studies in murine embryonic stem cells suggest that TET1 is a driver of demethylation at promoters while TET2 regulates demethylation at gene bodies [134]. TET2 expression decreases following implantation, although TET1 expression remains high until the early post-implantation stage, regulating key differentiation programs in the extraembryonic ectoderm and epiblast [119]. 

A number of knockout studies in mice to differentiate the roles of each TET enzyme during development have revealed interesting findings. A knockout of the TET1 catalytic domain results in mice that are both viable and fertile with a smaller body mass and litter [118,135]. However, the complete knockout of *Tet1* had a much more profound effect, with lethal embryonic deformities in the non-inbred mouse population [119]. *Tet2* knockout mice were also viable and fertile, although the loss of *Tet2* predisposes mice to the development of haematological malignancies [136,137,138,139]. While the double knockout of *Tet1/Tet2* is compatible with the development of viable and fertile mice, further developmental abnormalities are observed, including exencephaly and cerebral haemorrhages, and the majority of double knockout mice die within two days [135]. In contrast to the above, the homozygous knockout of *Tet3* causes neonatal lethality, signaling the indispensable role of the enzyme in early development [127]. Differential expression of TET enzymes in humans has also been observed with each TET protein being expressed at different stages of human spermatogenesis. Interestingly, TET expression was also found to positively correlate with fertilization rates, and patients with oligozoospermia and/or asthenozoospermia have lower TET expression in their sperm [140]. The general roles of each TET enzyme in development are summarised in Table 1.

A few studies have probed further into the specific interplay between vitamin C, TET activity and development. For example, DiTroia et al. employed a Gulo knockout mouse model to show that maternal vitamin C availability was required for the proper demethylation of DNA and optimal germ cell development in offspring [141]. While vitamin C deficient mothers were able to produce fertile offspring, these offspring exhibited an impaired ability to reproduce and showed a delayed onset of meiosis. Increased methylation was observed in vitamin C deficient embryos at sites of DNA involved in the regulation of meiosis and there was a significant decrease in 5hmC in liver and brain samples. A major finding from this study was the similarity between the transcriptomes of Tet1 knockout mice and germ cells in embryos from vitamin C deficient mothers. This finding implies that a deficiency in vitamin C during pregnancy phenocopies the loss of Tet1. 

Similarly, Kawahori et al. used a senescence marker protein-30/gluconolactonase (SMP30/GNL) knockout mouse model of vitamin C dependency to show that DNA hypermethylation occurred in the livers of offspring from vitamin C deficient mothers [142]. Over 400 genes were identified as being hypermethylated, including genes involved in fatty acid metabolism and glycerolipid metabolism. In contrast, the administration of high dose vitamin C to SMP30/GNL KO dams during the lactation period restored normal levels of methylation in the livers of these offspring and caused a significant elevation of liver 5hmC. These findings suggest a possible link between maternal vitamin C deficiency, compromised TET activity and the development of metabolic disorders in offspring.

One further point to consider when examining the interplay between vitamin C availability and TET activity is the possibility for the TET enzymes to regulate developmental processes via non-catalytic mechanisms [119]. The distinction between processes that are regulated via the enzymatic demethylation of methylcytosine vs. those which are regulated via protein-protein interaction is important–as vitamin C availability may not be relevant for the later mechanism.

## 10. The Histone Lysine Demethylases-Another Vitamin C-Dependent Class of Developmental Regulators

In addition to DNA methylation, the methylation of histone lysine residues is a key epigenetic mark which regulates the structure of chromatin and the accessibility of DNA for transcription [143]. Accordingly, histone lysine demethylase enzymes have an important function as epigenetic erasers and regulators of the epigenome. There are two broad classes of histone lysine demethylases. The FAD-dependent amine oxidases KDM1A/1B (LSD1/LSD2) utilise FAD to oxidise and hydrolyse methyl groups to formaldehyde and are not part of the 2-OGDD family [144]. The Jumonji C domain-containing histone lysine demethylases (KDM2–KDM7), however, catalyse the demethylation of lysine via the conserved 2-OGDD mechanism [145,146,147,148]. The enzymes KDM2–KDM7 deserve a mention in this paper due to their emerging regulatory roles in development and apparent dependency on vitamin C for optimal catalytic activity. These enzymes have been observed to regulate many facets of development such as sex differentiation [145,149], the reproductive system [150,151], neural development [152,153] and cardiac development [154]. They appear to require vitamin C for optimal activity [95,155]. This apparent requirement for vitamin C raises important questions. Consider the example of KDM5B (JARID1B). This enzyme has an emerging role in regulating the neural differentiation of embryonic stem cells [152] and a KDM5B knockout model has been shown to cause major embryonic defects such as defects in eye development, defective cranial nerves, exencephaly and post-natal death due to respiratory failure [153]. These findings are relevant given that the in vitro activity of KDM5B has been shown to be significantly compromised in the absence of vitamin C [156]. Given these intriguing observations, the roles of these enzymes in development and the nature of their vitamin C dependency are worthy of further consideration.

## 11. Epigenetic Programming and the Role of the TET Enzymes in Neurodevelopment

Epigenetic mechanisms are elemental to central nervous system (CNS) development and higher brain functions such as learning and memory [157,158]. An aberrant methylation landscape arising due to mutations or functional impairments in epigenetic proteins during development has been implicated in several neurodevelopmental disorders. Select examples include Tatton-Brown-Rahman, Rett, Fragile X, Angelman and Prader-Willi syndromes-all of which are characterised by intellectual disability and a range of behavioural and learning problems such as attention deficit hyperactive disorder (ADHD) and dyslexia [157,159,160]. Similar phenotypes are seen in circumstances where fetal nutrition is compromised-for example, maternal undernutrition and intrauterine growth restriction (IUGR) when infants are born with low birth weights, or preterm birth where infants do not receive their complete nutritional milieu in utero. These children are widely reported to exhibit neurodevelopmental deficits such as cognitive and learning difficulties and are at high risk of developing behavioural and emotional disorders [161,162,163,164,165,166]. The similarity in the phenotypic presentations of epigenetic and nutritional deficits highlights the importance of achieving an optimal nutritional environment for the fetus. It also alludes to the complex interplay between nutrition and epigenetics and the possible involvement of maternal micronutrient deficiencies in mediating adverse neurodevelopmental outcomes. 

In humans, the CNS begins to form 2–3 weeks post-conception and expands rapidly throughout the prenatal and perinatal periods with the brain reaching 80% of its adult volume by the age of two years [158,167,168,169]. The fetal brain begins as a small, smooth node that can be detected via functional magnetic resonance imaging (fMRI) from the ninth week of pregnancy [169]. As gestation proceeds, the brain undergoes striking structural changes with the folds designating distinct brain regions and the characteristic grooves (sulci) and ridges (gyri) on the cerebral surface becoming apparent [168,169]. These anatomical transformations reflect complex processes occurring at the cellular level under precise epigenetic control, namely the generation and coordination of distinct neural cell types [167,170].

## 12. Regulation of Neural Progenitor Cells and Neurogenesis

**Figure 4:**
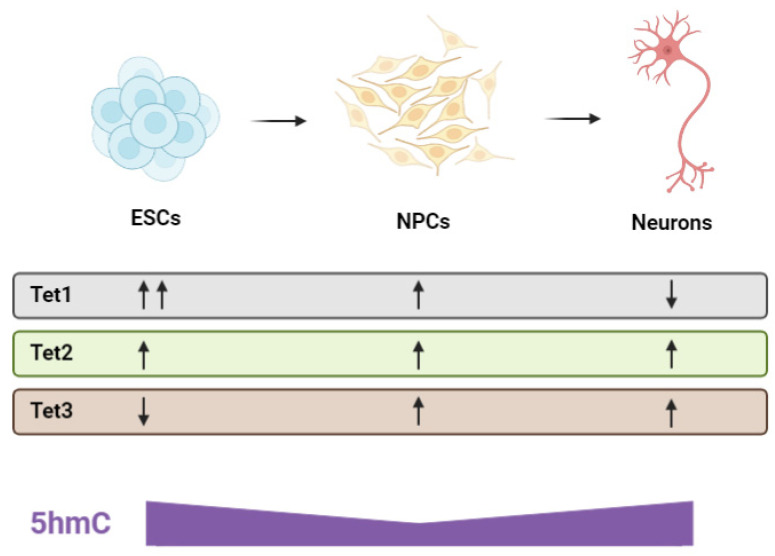
Schematic of TET Enzyme Expression and Corresponding 5hmC Levels during Neurogenesis. The three Tet enzymes have distinct and important roles in neurodevelopment. Tet1 is highly expressed in embryonic stem cells (ESCs) where it regulates the expression of pluripotency related genes. 5hmC levels in ESCs are correspondingly elevated. The expression of Tet1 decreases progressively during the ESC to neural progenitor cell (NPC) and NPC to neuron transition. Tet2 is basally expressed in all three cell types and is involved in controlling the balance between self-renewal and differentiation. Tet3 expression is negligible in ESCs but increases rapidly during the subsequent differentiation events, promoting the terminal differentiation of neurons. 5hmC levels are the lowest in the NPC population which reflects their partially-differentiated identity and transient state. In contrast, neurons exhibit high levels of 5hmC due to the dynamic nature of neuronal gene expression required for neuroplasticity. Figure created with Biorender.com accessed on 8 December 2021.

Early gestation is marked by neural progenitor cell (NPC) generation, proliferation, differentiation and cellular migration [167,170]. The formation of the neuroectoderm—A proliferative population of early NPCs—Represents the first step in mammalian CNS development [171,172]. Cells from the inner cell mass undergo sequential fate restriction by methylating pluripotency genes and demethylating genes specific to the neuroectoderm lineage in a spatiotemporal manner [172,173]. Research into embryonic stem cell (ESC) differentiation indicates that the TET enzymes play a pivotal role in this commitment of cells to the neural lineage. In cultured mouse cells, a global decrease in 5hmC occurs during the ESC to NPC transition [174]. This is accompanied by a decrease in *Tet1* expression with the paralog involved in maintaining the pluripotent state of ESCs (Figure 4) [93,175]. Select regions associated with neural functions also undergo de novo DNA hydroxymethylation [173,174] consistent with the understanding that NPCs have a limited differentiation capacity skewed toward the neural lineage. *Tet2*-knockout ESCs can successfully generate NPCs but exhibit hypermethylation at neural enhancers and delayed activation of neural-associated genes, highlighting the role of TET2 in neural differentiation [176]. Hypermethylation is also observed in the fetal brain of *Tet2*-knockout mice [177]. 

The expression of *Tet3* increases rapidly during ESC and NPC differentiation [178]. In *Tet3*-knockout ESCs, self-renewal appears normal and cells can differentiate into NPCs but these rapidly undergo apoptosis, leading to an insufficient progenitor pool [178]. Notably, vitamin C appears to have a role in neurodevelopment at the cell culture level. The addition of vitamin C to ESC cultures, at concentrations similar to those found in the CSF, induces the expression of genes such as *NeuroD*, *Notch,* and *BMP2* that stimulate neural differentiation [179,180]. The expression of genes involved in neural migration such as *DCX* (Doublecortin) also become upregulated [180]. These effects are not replicated through treatment with other antioxidants like vitamin E, supporting the theory that vitamin C directs ESC differentiation through a TET enzyme-mediated mechanism [179].

Once a sufficient pool of neuroectoderm progenitors is established, the population of cells structurally transforms into the neural plate, the borders of which fold up and eventually converge to form the neural tube. Neurogenesis then proceeds in the walls of the neural tube to grow the brain and spinal cord [168,173]. Neurogenesis is the process by which terminally differentiated neurons are generated from NPCs and then migrate out to their final locations in the CNS to be integrated into synaptic circuits [181]. The process occurs primarily in utero during the growth and development of nervous tissue but also occurs in regions of the adult brain, particularly following injury [173]. Hahn et al. have mapped patterns of 5mC and 5hmC during neurogenesis in the fetal mouse brain and observed that 5hmC levels increase during the NPC to mature neuron transition. Further, 5hmC was enriched at genes associated with mature neuronal functions [182].

Investigation of the distinct functions of the individual TET proteins during neurogenesis has been carried out in the adult mouse brain and derived NPCs. A specific role for *Tet2* in regulating the balance between NPC self-renewal and neuronal differentiation has been recognised. The knockout of *Tet2* leads to increased NPC proliferation and a significant reduction in the number of newly-born neurons indicating an impaired differentiation capacity [183,184]. This is accompanied by a reduction in *DCX*-expressing neurons suggesting defective migration [180]. In contrast, the depletion of *Tet1* leads to decreased NPC proliferation with hypermethylation and downregulation of genes involved in progenitor maintenance [185]. In *Tet3*-knockout NPCs, the pluripotency genes *Oct4* and *Nanog* become de-repressed [111] and fewer progenitors differentiate into neurons [178] demonstrating that TET3 maintains the partially-differentiated identity of NPCs and along with TET2, promotes the terminal differentiation of NPCs into mature neurons. These findings highlight that all three TET enzymes play a role in regulating the NPC pool and the proper differentiation of neurons. Studies on the effects of dietary vitamin C depletion during prenatal brain development have produced remarkably similar results to the *Tet*-knockout studies described above. As mentioned previously, suboptimal vitamin C status in prenatal (beginning in the first trimester until birth) [12] or early postnatal life (6–7 days old) [13] substantially impairs hippocampal development in guinea pigs. When a partial deficiency was imposed prenatally–achieved by reducing the vitamin C content in the diets of pregnant dams–offspring exhibited a significant decrease in overall hippocampal volume and the total number of new cells migrating within the hippocampus was reduced. These impairments in postnatal hippocampal development persisted as offspring aged and could not be alleviated by dietary vitamin C repletion from the first week of life [12]. These findings could reflect the dependency of the TET enzymes on vitamin C for optimal function during critical stages of early neurodevelopment. A depiction of Tet expression levels during neurogenesis and corresponding 5hmC levels is highlighted in Figure 4 below.

The knockout of *Tet1* does not completely abolish neurogenesis or cause neuroanatomical defects [185,186]. However, the double knockout of *Tet1*/*Tet2* or *Tet1*/*Tet3* respectively causes exencephaly and holoprosencephaly in some embryos [135,187]. This indicates that while each paralog has a unique function in neurodevelopment, there is also a level of redundancy. Yet, this redundancy may only be partial as the complete loss of *Tet3* has been reported to cause neonatal lethality in mice [188]. 

## 13. Synaptogenesis, Myelination and TET Function

The latter half of pregnancy is marked by synaptogenesis and myelination both in humans and precocial species such as guinea pigs [189]. This contrasts with altricial species such as rats and mice where the establishment of functional neural circuits occurs predominantly in the weeks following birth [189]. During synaptogenesis, neurons assemble and communicate to form the intricate and wide-reaching ‘connectome’-the brain’s superhighway of information, that coordinates all functions from involuntary actions like breathing and digestion to conscious thought, reasoning and emotion [167,169]. Throughout this elaborate cortical organisation process, neural networks are continually refined and remodelled. Excessive NPCs and synaptic connections must also be scaled down and these are selectively eliminated via apoptosis [167,168]. The apoptotic process is tightly regulated, as inappropriate timing or imbalances in the formation vs. deletion of synapses may lead to ineffective neural circuitry and produce negative cognitive outcomes later in life [158,190]. This capacity of the nervous system to reorganise neural circuits—referred to as neuroplasticity—constitutes the molecular basis of learning and memory. Through long-term potentiation (LTP) and long-term depression (LTD), synaptic connections are, respectively, strengthened or weakened, in an activity-dependent manner [190,191]. In the postnatal brain, the TET enzymes have been shown to act as synaptic activity sensors through modulating levels of 5mC and 5hmC in response to neuronal firing [185,186,192,193,194,195]. This remodelling of neuronal epigenomes is required for the correct expression of genes involved in memory formation and consolidation such as *Arc* and *Bndf*, with the TETs specifically implicated in the regulation of spatial memory and fear memory extinction [185,186,192,193,194,195,196]. In the fetal brain, neural apoptosis can also be regulated by levels of synaptic activity [167], highlighting a potential role of the TET enzymes as synaptic activity sensors, in the proper development of neural circuitry in utero.

The formation of the myelin sheath, mediated by Schwann cells in the periphery and oligodendrocytes in the CNS, is a critical step in the neuronal maturation pathway and is required for synaptic transmission. In the 1980s, Eldridge et al. revealed that adding vitamin C to co-cultures of Schwann cells and dorsal root ganglia facilitated the formation of myelin sheaths [197]. Experiments using co-cultures of oligodendrocyte precursor cells (OPC) and neurons have produced similar results, with vitamin C promoting rapid sheath development [198]. However, the exact mechanism of action has remained elusive until recently. Like most cell types in the body, vitamin C enters myelinating cells via the SVCT2 transporter [198,199]. In mice haploinsufficient for the *SLC23A2* gene that encodes the SVCT2 membrane transporter protein, vitamin C fails to accumulate within Schwann cells and this results in a thinner layer of myelination on axon fibres and impaired synaptic transmission [200]. This finding was pivotal in directly establishing that intracellular, rather than extracellular, vitamin C directs myelination in vivo. This supports the theory that vitamin C exerts its neurogenic-positive effects through epigenetic interactions with DNA at the intracellular level. A recent study has shown that vitamin C exposure produces a global increase in 5hmC levels and upregulates the expression of several pro-myelinating genes in Schwann cells [201], supporting a role for the TET enzymes in directing myelination.

## 14. Impact of *Tet* Mutations in Neurodevelopment

Further evidence validating the importance of the TETs in neurodevelopment and brain function has come from the recent discovery of *Tet3* mutations in a cohort of families with the common phenotypic features of intellectual disability, growth and craniofacial abnormalities, and autistic traits [202]. While there was some variance in mutation type and in inheritance patterns across families, all affected individuals exhibited a general deficiency in TET3 function [202]. The clinical characteristics of affected individuals closely resemble what is seen in patients with other disorders of the epigenetic machinery like Tatton-Brown-Rahman syndrome-caused by mutations in *DNMT3A* [198,199,203]. These findings emphasise the importance of proper epigenetic programming in human neural development. A summary of the TET enzymes and their roles in neurodevelopment is included below in Table 2.

## 15. Translational Animal Models for Dietary Vitamin C Studies

Given the importance of maternal nutrition on epigenetic programming and health outcomes in later life, it is imperative that we develop the right paradigm to accurately study the effects of diet on early development. Numerous epidemiological studies have indicated that dietary variations during development can impact on health outcomes [204,205,206]. However, dietary intervention studies in humans are complicated by differences in lifestyle that result in a plethora of uncontrolled variables. In addition, estimates of nutritional status based on dietary intake by human recall are highly unreliable, and seasonal effects on produce, varying food storage and preparation techniques add complications to any studies [3,207,208]. Access to organ and tissue samples further adds to the challenge of human studies. In contrast, animal models provide a valuable experimental opportunity to overcome these limitations and study controlled dietary manipulations in vivo. 

While rats and mice are convenient laboratory animals, they have the ability to endogenously synthesise vitamin C, meaning that mutant knockout animal models need to be established for dietary vitamin C studies [209,210]. The guinea pig, however, is a relatively low-maintenance laboratory animal that can be housed in high numbers and is also a natural model of diet-induced vitamin C deficiency. Like humans, guinea pigs lack the capability to synthesise endogenous vitamin C due to a defective gulonolactone oxidase gene [1,2]. Another useful feature of the guinea pig experimental model is that the conditions for regulating vitamin C status in guinea pigs are now well-documented in the literature. A diet containing 100 mg vitamin C/kg of feed successfully induces a non-scorbutic vitamin C deficiency in animals of all ages [13,26,211], including during pregnancy [9,10,12].

The pregnant guinea pig is in itself a well-established model that closely resembles human pregnancy and prenatal development, as previously reviewed [189]. The structure (i.e., haemomonochorial) of the guinea pig placenta is analogous to that of the human with deep trophoblast invasion, and similarities in circulating progesterone concentrations across gestation and at the time of parturition [212,213,214]. The timing of key stages of fetal development is also comparable to humans, with the full maturation of the pup brain and other organs occurring before birth, unlike in altricial species such as rats and mice where this occurs postnatally [189,215]. The guinea pig has a relatively long gestation at 69–71 days (19–21 days for other rodents), which offers a distinct advantage in terms of investigating prenatal challenges. Not only does the longer gestation period allow for better resolution of developmental plasticity [189], it also provides sufficient time for dietary manipulations such as maternal vitamin C depletion that may take a number of days or weeks to become properly established [26,216], to be investigated. The clear parallels between vitamin C metabolism, pregnancy, and fetal development of the human and the guinea pig support the use of this species as a relevant model to explore the neurodevelopmental impact of low maternal vitamin C during pregnancy.

## 16. Conclusions

The evidence is now conclusive—in utero experiences, particularly fetal nutrient availability as mediated by the maternal diet, can translate to phenotypic variation and future disease susceptibility that extends well into adulthood and even subsequent generations. The underlying biological mechanisms are indeed complex but huge advancements have been made with the advent of epigenetics. The TETs are a family of epigenetic enzymes that are highly expressed during mammalian development. As epigenetic erasers that promote the hydroxylation and demethylation of DNA, the enzymes function to modulate gene expression and coordinate complex processes such as cellular differentiation. A dozen years on from their initial discovery, there is a rapidly expanding body of literature that highlights the important roles of the TET enzymes in various aspects of neural physiology. The TET enzymes regulate NPC biology, neurogenesis, higher brain functions and behaviours, and have recently been implicated in the aetiology of neurological disorders. The dependency of the TETs on vitamin C for optimal catalytic activity becomes particularly relevant when considering the impact of nutrition on epigenetics in development. Future work examining the relationship between maternal vitamin C status and TET-mediated processes during development in utero will not only augment what is known about these prominent epigenetic enzymes but will greatly enhance our understanding of metazoan brain development and how early-life nutrition can program long-term cognitive outcomes. These questions are of great importance given the prevalence of hypovitaminosis C in pregnant women worldwide.

## Figures and Tables

**Table 1:** The TET Enzymes, Their Dependency on Vitamin C, and Their General Role in Development.

Enzyme	Effect of Vitamin C	Role in Development
TET1	Increased activity in vitro [102,112] and in cell culture [101,102,114,115,116,117]	Highly expressed in mouse embryonic stem cells [93,118]. Expressed in the developing brain [119]. High expression in the early post-implantation stage, regulates differentiation in the extraembryonic ectoderm and epiblast [119].
TET2	Increased activity in vitro [101,112], in cell culture [101,102,114,117,120], in mice [121,122], and in a clinical setting [123]	Regulation of haematopoiesis, mutations are a driver of leukaemia and other haematopoietic pathologies [95,121,124,125,126]. Expressed in the developing brain [119].
TET3	Increased activity in vitro [112], in cell culture [114,117], and mice [121]	Indispensable for proper development as knockout results in embryonic lethality [127]. Mediates cell-fate decisions by inhibiting Wnt signaling, regulates neural and mesodermal cell fate [128]. Highly expressed in oocytes and zygotes [127,129,130]. Involved in the demethylation of zygotic paternal DNA following fertilization [127]. Expressed in the developing brain [119].

**Table 2:** The TET Enzymes and Their Roles in Neurodevelopment and Brain Function.

Enzyme	Roles in Neurodevelopment and Brain Function
TET1	Maintains the pluripotent state of ESCs [93], promotes NPC proliferation and maintenance [185]. Expression becomes downregulated in response to neuronal firing [185,186,192]. Knockout leads to impairments in spatial and short-term memory [185], defective fear memory extinction and enhances LTD [186].
TET2	Enhances and skews ESC differentiation toward to the neural lineage [176], controls NPC proliferation and promotes terminal neuronal differentiation [183,184]. Combined loss with *Tet1* can cause exencephaly [135].
TET3	Preserves the partially-differentiated identity of NPCs [111] and promotes terminal neuronal differentiation [178]. Expression becomes upregulated in response to neuronal firing [193,195]. Combined loss with *Tet1* can cause holoprosencephaly [187]. Deficiency in TET3 function has been linked to a familial intellectual disability disorder in humans [202].

Findings are in mice or mouse-derived cells unless otherwise stated.
